# Asthma and pregnancy: profile of the patients cared at Hospital Geral de Nova Iguaçu (HGNI), NOVA IguaÇu, Brasil

**DOI:** 10.1186/1939-4551-8-S1-A99

**Published:** 2015-04-08

**Authors:** Sylvia Gabriella Maia Araújo, Raphael Coelho, Aniele Soares Moritz, Gustavo Henrique De Martin Ballini, Gisele Cardoso Nery, Luiz Gustavo Bernardo De Oliveira, Daniella Mouta Dos Santos Silva, Sérgio Duarte Dortas

**Affiliations:** 1 Universidade Iguaçu, Brazil; 2 Hospital Universitário Clementino Fraga Filho Hucff-Ufrj, Brazil

## Background

Asthma is a chronic disease, that can affect women during pregnancy, with potential risk for maternal and fetal life.

We aim to describe the profile of the pregnant women, assisted in an Emergency Department, presenting bronchospam (ICD10: J45 and J42) from January 2008 to March 2014.

## Methods

A descriptive cross-sectional study with retrospective data collection from pregnant women with ICD10 J45 and J42 in the emergency service of HGNI. Parameters analyzed were: age, race, type of attacks, medications used, period of hospitalization, complications and gestational age.

## Results

Data of 13 patient were collected, with average age of 26 years old (14-35 years old). Considering race, most were brown (69%), black being the minority (8%). As for the classification of the attacks, there was a highest prevalence of mild to moderate attacks (54%), followed by 38% of severe attacks and 8% respiratory arrest imminent. The medication used in 100% of the patients was injected corticosteroid, associated with inhaled beta-2-agonist in 85% of the cases. Injectable aminophyline was used in 3 cases. Complications were present in 61% of the pregnant women, being pneumonia the most prevalent (54%), and just one patient with preeclampsia (Specific Gestacional Hipertensive Disease). The average gestacional age was 25 weeks (21-32 weeks).

## Conclusions

Our series show that asthma is a reality in the course of the pregnancy period and that should be better evaluated due to the severity of the attacks that led the patients to the emergency services. That confirms the findings of other studies in that the presence of the attacks more evident in the third quarter. The large percentage of complications displays the potential of severity that the disease can reach. It is important to point out that asthma is a disease that is present in pregnancy and can bring risks for mother and fetus, so it should have a disease control program before pregnancy due to the risk of its aggravation in the course of the gestational period, as well as the precocious detection of the disease that appears during the pregnancy to avoid complications. The educational approach to asthma during pregnancy should include complementary diagnosis as much as the medications to be used, with focus on the primary attention.

